# Peripheral Neuropathy Presents Similar Symptoms and Pathological Changes in Both High-Fat Diet and Pharmacologically Induced Pre- and Diabetic Mouse Models

**DOI:** 10.3390/life11111267

**Published:** 2021-11-19

**Authors:** Julia Jaroslawska, Agnieszka Korytko, Kamila Zglejc-Waszak, Tomasz Antonowski, Andrzej S. Pomianowski, Krzysztof Wasowicz, Joanna Wojtkiewicz, Judyta K. Juranek

**Affiliations:** 1 Department of Biological Functions of Food, Institute of Animal Reproduction and Food Research, Polish Academy of Sciences, 10-748 Olsztyn, Poland; 2 Department of Human Physiology and Pathophysiology, School of Medicine, University of Warmia and Mazury, 10-082 Olsztyn, Poland; agnieszka.korytko@uwm.edu.pl (A.K.); kamila.zglejc@uwm.edu.pl (K.Z.-W.); tomasz.antonowski@uwm.edu.pl (T.A.); joanna.wojtkiewicz@uwm.edu.pl (J.W.); 3 Department of Internal Medicine, Faculty of Veterinary Medicine, University of Warmia and Mazury, 10-718 Olsztyn, Poland; andrzej.pomianowski@uwm.edu.pl; 4 Department of Pathophysiology, Faculty of Veterinary Medicine, University of Warmia and Mazury, 10-718 Olsztyn, Poland; krzysztof.wasowicz@uwm.edu.pl

**Keywords:** diabetic peripheral neuropathy, sciatic nerve, diabetic mouse models, streptozotocin, high-fat diet

## Abstract

The objective of the study was to compare the effects of experimentally induced type 1 or type 2 diabetes (T1D or T2D) on the functional, structural and biochemical properties of mouse peripheral nerves. Eight-week-old C57BL/6 mice were randomly assigned into three groups, including the control (CTRL, chow-fed), STZ (streptozotocin (STZ)-injected), and HFD (high-fat diet (HFD)-fed) group. After 18-weeks of experimental treatment, HFD mice had higher body weights and elevated levels of plasma lipids, while STZ mice developed hyperglycemia. STZ-treated mice, after an extended period of untreated diabetes, developed motor and sensory nerve conduction-velocity deficits. Moreover, relative to control fibers, pre- and diabetic axons were lower in number and irregular in shape. Animals from both treatment groups manifested a pronounced overexpression of nNOS and a reduced expression of SOD1 proteins in the sciatic nerve, indicating oxidative–nitrosative stress and ineffective antioxidant protection in the peripheral nervous system of these mice. Collectively, STZ- and HFD-treated mice revealed similar characteristics of peripheral nerve damage, including a number of morphological and electrophysiological pathologies in the sciatic nerve. While hyperglycemia is a large component of diabetic neuropathy pathogenesis, the non-hyperglycemic effects of diabetes, including dyslipidemia, may also be of importance in the development of this condition.

## 1. Introduction

Diabetic peripheral neuropathy (DPN) affects nearly 50% of adults with diabetes, which makes it one of the most common complications of this condition [1]. DPN is a neurodegenerative disorder that preferentially targets sensory and autonomic axons, subsequently damaging motor nerve terminals, however the latter are most often affected to a lesser degree [2]. The disorder leads to a loss of a sensory function resulting in so-called neuropathic pain and is associated with substantial morbidity.

Peripheral nerves are the most susceptible tissues to pathological changes due to hyperglycemia. The mechanism underlying neuropathy in diabetes remains unknown, but it has been established that RAGE (receptor for advanced glycation end products) might play a role in its pathogenesis [3]. It has been speculated that long-term hyperglycemia could cause RAGE-related inflammation and oxidative stress. RAGE, by its binding to pro-inflammatory ligands such as high-mobility group protein (B)1 (HMGB1) and S100B, causes the activation of nuclear transcription factors such as NF-κB, STAT and JKN [3]. RAGE can also contribute to the progress of several neurodegenerative diseases: Parkinson’s, Huntington’s, Alzheimer’s and amyotrophic lateral sclerosis (ALS) [3].

T1D is a chronic disease that is caused by the autoimmune destruction of insulin-producing β cells in the pancreas, leading to insulin deficiency, whereas, in T2D, both decreased β-cell insulin secretion and peripheral insulin resistance play a parallel role. Despite T1D and T2D having different pathophysiology, most of the complications are similar. The link between diabetes and DPN could be a result of a long-term, continued elevation of blood glucose levels; however, studies show that the molecular and biochemical mechanisms leading to the development of this condition could be more versatile and complex [4,5,6]. Growing evidence indicates that the pathological changes observed in diabetic sciatic nerves might result from excessive protein glycation, increased oxidative stress, inflammation or abnormalities in insulin secretion and/or activation. Excessive lipids circulating in the blood may also play a relevant role in the development of this condition [7,8,9]. 

The present study was undertaken to observe the electrophysiological, biochemical and structural changes in pre-diabetic and diabetic peripheral nerve of mice following STZ administration or HFD feeding, taking into consideration potential similarities or differences in evoked pathologies, originating from different modes of diabetes induction in mice.

## 2. Materials and Methods

### 2.1. Animals

Breeding pairs of C57BL/6 wild-type mice were bred at the Animal Facility at the University of Warmia and Mazury. Mice were maintained in ventilated rooms under a standard-day photoperiod (12:12 h light-dark period, lights on from 700 to 1900 h at 21 °C in with free access to food (Labofeed B) and water. At 56 days of age mice were randomized into three experimental groups (*n* = 8 per group) and treated with: (i) STZ (intraperitoneal injection of 50 mg/kg STZ diluted in PBS for 5 days)), (ii) PBS (intraperitoneal injection of equal volume and period of treatment) or (iii) fed HFD (ssniff Spezialdiäten GmbH, Surwit with sucrose E15772-34) ad libitum for 4.5 months (18 weeks). At 2- and 4.5 months post STZ administration/HFD introduction, the mice underwent electrophysiological tests to assess nerve-conduction velocity (NCV). After 4.5 months, mice were anesthetized with a mixture of ketamine (300 mg/kg) and xylazine (30 mg/kg). Blood samples were collected into EDTA-coated Eppendorf tubes and centrifuged at 2500× *g* for 10 min at 8 °C. Plasma was stored at −20 °C. Sciatic nerves were immediately frozen in liquid nitrogen and stored at −80 °C for subsequent analyses. All experiments were approved by the local ethics committee of the University of Warmia and Mazury (no. 57/2019).

### 2.2. Phenotypic Assays

Body weight (BW) was measured weekly. To determine the diabetes status of animals with STZ- induced diabetes, blood glucose was measured 7 days after the first STZ injection using an Accu-Chek glucometer (Roche, Switzerland) and then every three weeks, in mice from each group, for the duration of the experiment. Among STZ mice, only animals with a glycemia of 13 mmol/L (260 mg/dL) were considered diabetic and used for further experiments.

### 2.3. Plasma Assays

Blood lipids: total cholesterol (TC), high-density lipoprotein (HDL) cholesterol, and triglyceride (TG) concentrations were assessed in plasma by using an enzymatic colorimetric procedure with a Pentra C200 Chemistry Analyzer (Horiba Ltd., Kyoto, Japan). Low-density lipoprotein (LDL) cholesterol was calculated by the Friedewald formula (TC minus HDL-cholesterol minus TGs/5 in mmol/L). The circulating isoform of RAGE was measured in plasma using commercially available Quantikine ELISA Mouse RAGE Immunoassay (MRG00, R and D Systems, Minneapolis, MN, USA).

### 2.4. Western Blot Analysis

Proteins from whole, unilateral sciatic nerve were extracted using an All Prep DNA/RNA/Protein Mini (Qiagen, Germany). Protein concentration was determined with Direct Detect^®^ Infrared Spectrometer (Merck Millipore, Germany). Proteins (40 µg) were separated on 15-well 4–15% Mini-PROTEAN^®^ TGX™ Precast Protein Gels (Bio-Rad, Hercules, CA, USA) and transferred onto a nitrocellulose membrane using the semi-dry system (Trans-Blot Turbo Transfer System, Bio-Rad). The membrane was blocked for 5 min in EveryBlot Blocking Buffer (Bio-Rad) and incubated overnight at 4 °C with primary antibodies diluted in SignalBoost™ Immunoreaction Enhancer solution (Merck Millipore). The primary antibodies used in the Western blot analyses were as follows: anti-SOD1 (1:2000, Abcam, ab13498); anti-S100B (1:1000, Abcam, ab52642); anti-HMGB1 (1:1000, Abcam, ab18256); anti-nNOS (1:2000, Abcam, ab1376); anti-RAGE (1:1000, Abcam, ab37647) and anti-β-ACTIN (1:1000, Abcam, ab6276). After four washes in PBS with 0.1% Tween-20, the membrane was incubated for 2 h at RT with the corresponding fluorescence-labelled secondary antibody (IRDye800 and IRDye700, LI-COR Biosciences, Lincoln, NE, USA) diluted in SignalBoost™ Immunoreaction Enhancer solution (Merck Millipore). The bands were visualized with ChemiDoc Imaging Systems (Bio-Rad). Images were quantified densitometrically with ImageJ Software 1.50i (Wayne Rasband, Bethesda, MD, USA) and compared to experimental condition after normalization to the total amount of protein in a sample.

### 2.5. Immunohistochemical (IHC) Staining 

Explants of sciatic nerves were transferred for 12 h to −20 °C, cut on a CM3050 cryostat (Leica Microsystems Inc., Buffalo Grove, IL, USA) into 8-µm sections and mounted onto poly-l-lysine-coated glass microscope slides (Menzel-Glaser, Braunschweig, Germany). Immunohistochemical analysis was performed as described in a protocol of VECTASTAIN^®^ ABC-HRP Kit, Peroxidase (Rabbit IgG, PK-4001; Vector Laboratories, Burlingame, CA, USA). Tissue sections were incubated with 2.5% horse normal serum (Vector Laboratories) for 1h at RT. Subsequently, the slides were washed for 5 min in PBS, air-dried and incubated with rabbit primary antibodies, the same as those used in the immunoblotting analysis, i.e., anti-β-ACTIN (1:100), anti-S100B (1:100), anti-HMGB1 (1:200), anti-RAGE (1:50), anti-SOD1 (1:50) and anti-nNOS (1:50), diluted in 0.1% BSA at 4 °C, overnight. For negative controls, tissue slices were incubated with 2.5% horse normal serum instead of the primary antibodies. After overnight incubation with primary antibodies, sections were washed in PBS 3× for 5 min each and incubated with corresponding secondary antibodies (commercially diluted, Vector Laboratories) for 1 h at RT. Sections were immersed in 3,3 diaminobenzidine tetrahydrochloride (DAB, Dako, Santa Clara, CA, USA) followed by hematoxylin staining (Aqua-Med, Poland). Subsequently, sections were dehydrated in graded series of ethanol (70, 90, 100%), cleared in xylene, and mounted with DPX (Sigma Aldrich, Saint Louis, MO, USA). The labelled tissues were photographed using a C-5060 Camera (Olympus, Tokyo, Japan) mounted on a light microscope (CH30/CH40, Olympus). Images were taken with magnification of 400×, and areas of staining were determined with Cell^F software (Olympus).

### 2.6. NCV Analysis

All animals were intraperitoneally anesthetized with a mixture of ketamine (100 mg/kg) and xylazine (10 mg/kg). During anaesthesia, body temperature was maintained at 37 °C using a warming pad and eye drops were used to prevent eye dryness. Needle electrodes (Rhythmlink International LCC, Columbia, SC, USA) were cleaned with 70% ethanol between the animals. The studies were performed using a Nicolet Viking Quest Apparatus and Nicolet Viking, version X computerized system (CareFusion, San Diego, CA, USA). Measurement of NCV was performed as described by Schulz et al. (2014) [10], with modifications, according to the Diabetes Complications Consortium Nerve Conduction Protocol (www.diacomp.org accessed on 16 October 2019).

For motor NCV (MNCV) studies, the sciatic nerve was stimulated twice (proximal and distal stimulation), recording from the same site. Evoked compound muscle action potentials for both stimulations were recorded from the gastrocnemius muscle using needle-recording electrodes, with the reference electrode in the ankle tendon. For proximal stimulation, the active electrode was placed in the popliteal fossa, with the reference electrode 3–5 mm distally. For distal stimulation, the active electrode was placed on the upper thigh near the midline at the sciatic notch, with the reference electrode 3–5 mm distally. Amplitudes were measured in mV, and latencies in ms. MNCV was calculated by dividing the distance between electrodes placed on the upper thigh near the midline at the sciatic notch and electrodes placed in the popliteal fossa (measured with a fine calliper) by the difference in latency during stimulation at the sciatic notch compared with that obtained during popliteal fossa stimulation, to yield a velocity in m/s. A square wave stimulus pulse (0.02 ms) at a very low amperage (1.9–5.7 mA, average 2–4 mA) was delivered using a bipolar Nicolet Viking stimulator probe (Model number S403), through attached subdermal needle electrodes (Nicolet Biomedical disposable SS-Subdermal Needles). 

For SNCV, the sural nerve was stimulated orthodromically using needle electrodes placed in the fourth toe of the foot, with recording via needle electrodes in the gastrocnemius muscle (as described for MNCV). SNCV was calculated by dividing the distance between the stimulating and recording electrodes by this latency. The amplitudes were measured in μV, and the latencies in ms. 

### 2.7. Morphometric Analysis

Morphometric analysis of the sciatic nerves was performed as described previously by Juranek et al. (2012), with modifications. The collected tissues were immersed in 4% paraformaldehyde in PBS, pH 7.4 overnight, afterwards rinsed with PBS 3× for 10 min. Tissues were then post-fixed in 2% osmium tetroxide (Sigma Aldrich) for 2 h and rinsed 3× for 5 min with distilled water. Next, tissues were dehydrated in a graded series of alcohol (in PBS) for 2 h each (30, 50, 70, 90, 100%), cleared in xylene, paraffined and cut into 1.5-μm slices using a microtome CM3050 (Leica) and mounted onto poly-L-lysine-coated glass slides (Menzel-Glaser). The slides were kept at 30 °C overnight, cleared in xylene, hydrated in graded series of alcohol (100, 90, 70, 50, 30%) and stained with 1% toluidine blue (Sigma Aldrich) in PBS for 15 min. Following the staining, tissues were rinsed with PBS 3× for 10 min, dehydrated in ethanol, cleared in xylene and mounted with DPX (Sigma Aldrich, USA). The labelled tissues were photographed using a C-5060 Camera (Olympus) mounted on a light microscope (CH30/CH40, Olympus). Pictures were taken with 200× magnification, and areas of staining were determined with Cell^F software (Olympus). The full-size cross-sections of distal sciatic nerve fascicule from each group were analysed using ImageJ software. The myelinated axon quantification was performed for four technical replicates per each of four tissue samples within one animal per group, as described by Hunter et al. (2007).

### 2.8. Statistical Analysis

Statistical analysis of all parameters was carried out with GraphPad Prism Software (Version 7.0a Graph Pad Software Inc., La Jolla, CA, USA). The normality of the data was verified with the Shapiro–Wilk test. All data sets were analyzed using analysis of variance (one-way ANOVA). Where *p*-values for the ANOVA indicated a significant effect of any factor, a Tuckey’s multiple comparisons test was performed post-hoc to determine which groups in the ANOVA differed from each other. Data are expressed as means ± SEM. Differences between the means at * *p* < 0.05, ** *p* < 0.01, *** *p* < 0.001, **** *p* < 0.0001 were considered significant. 

## 3. Results

### 3.1. STZ Injections and HFD Administration Induce Diabetes-Related Phenotypic Characteristics in Mice

**Figure 1:**
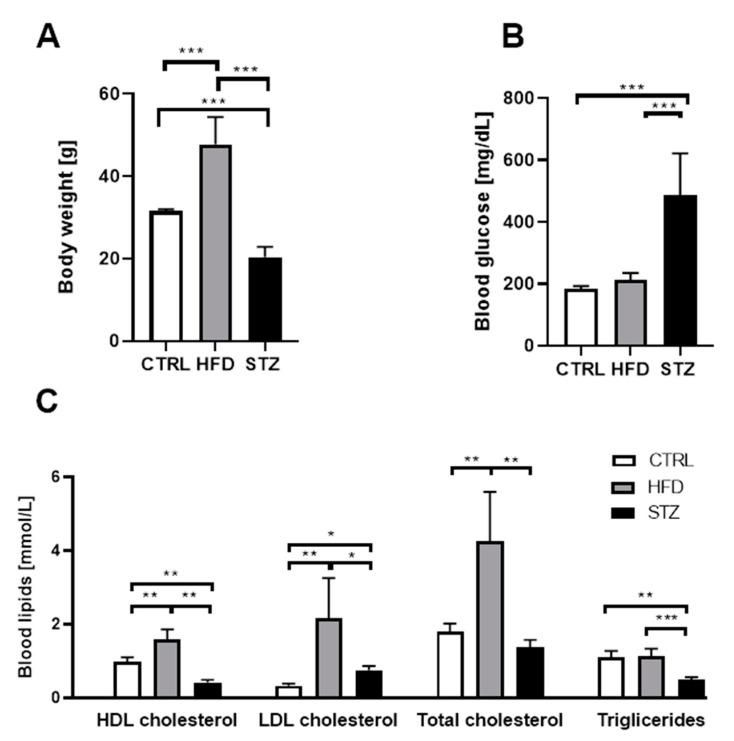
STZ injections and HFD administration induce diabetes-related phenotypic characteristics in mice. Body weight (**A**), blood glucose (**B**) and blood lipids (**C**) changes in mice 4.5 months after STZ administration/HDF introduction. Data are expressed as means ± SEM. * *p* < 0.05; ** *p* < 0.01; *** *p* < 0.001; *n* = 8.

Long-term HFD feeding resulted in a significant increase in mice BW, as compared to other groups (47.71 ± 2.35 vs. 20.39 ± 0.87 g in STZ and 31.57 ± 0.47 in CTRL group, *p* < 0.001) (Figure 1A). Multiple STZ injections were associated with substantial weight loss in mice (*p* < 0.001). Compared with HFD-fed and control mice, mice with STZ-triggered T1D had markedly higher blood glucose level (487.5 ± 47.39 vs. 213.5 ± 7.91 and 183.65 ± 9.37 mg/dl, respectively, *p* < 0.001) (Figure 1B). Eighteen weeks of HFD feeding was not enough to induce hyperglycemia. However, in comparison with CTRL and STZ-treated mice, HFD-fed mice had significantly elevated levels of blood LDL cholesterol (2.16 ± 0.45 vs. 0.33 ± 0.02, *p* < 0.01 and 0.75 ± 0.11 mmol/L, *p* < 0.05; respectively), TC (4.25 ± 0.55 vs. 1.80 ± 0.09 and 1.38 ± 0.18 mmol/L, respectively, *p* < 0.01), and HDL cholesterol (1.58 ± 0.11 vs. 0.97 ± 0.05 and 0.41 ± 0.07 mmol/L, respectively, *p* < 0.01) (Figure 1C). 

### 3.2. STZ-Treated Mice Exhibit Deficits in Both Sensory and Motor NCV after 4.5 Months of Diabetes

**Figure 2:**
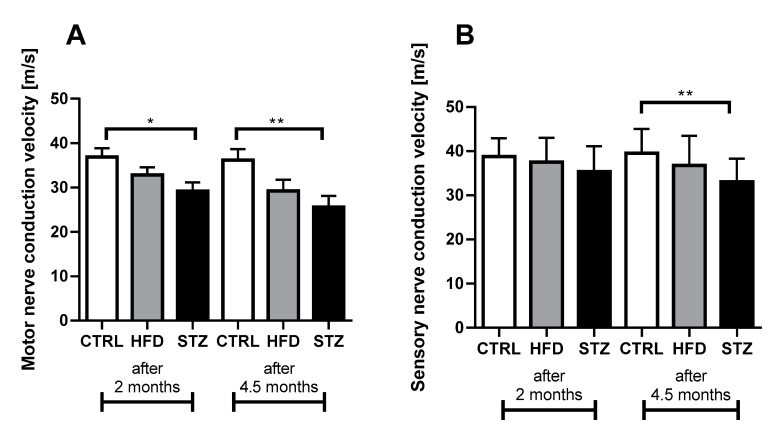
STZ-treated mice exhibit deficits in both sensory and motor NCV after 4.5 months of diabetes. Motor (**A**) and sensory (**B**) nerve conduction velocity (MNCV and SNCV, respectively) measured 2 and 4.5 months after STZ administration/HFD introduction. Data are expressed as means ± SEM. * *p* < 0.05; ** *p* < 0.01; MNCV tests: *n* = 8, SNCV tests: *n* = 8.

As compared to CTRL, MNCV values were significantly decreased in STZ mice, both at 2 (36.86 ± 1.97 vs. 29.22 ± 1.95 m/s, respectively, *p* < 0.05) and 4.5 (36.15 ± 2.49 vs. 25.63 ± 2.45 m/s, respectively, *p* < 0.01) months after STZ administration (Figure 2A). In comparison with controls, SNCV already values tended to be reduced at 2 months after STZ administration/HFD introduction in both animal groups (Figure 2B); however, the significant decline in this parameter was observed only in STZ mice at the end of the experiment (39.52 ± 1.47 vs. 33.00 ± 1.47 m/s, respectively, *p* < 0.01).

### 3.3. STZ Injections and HFD-Feeding Lead to Morphometric and Morphological Changes in Mouse Peripheral Nerves

**Figure 3:**
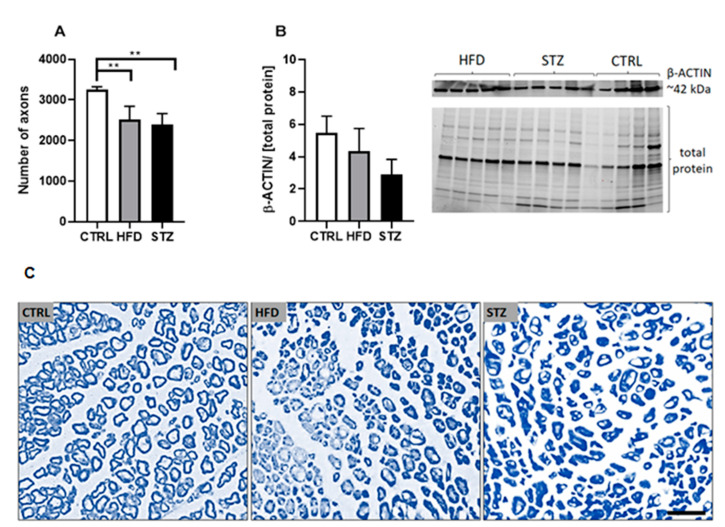
STZ injections and HFD-feeding lead to morphometric and morphological changes in mouse peripheral nerves. Number of axons per semithin whole nerve cross-section measured in mice 4.5 months after STZ administration/HFD introduction (**A**), *n* = 4. β-ACTIN expression measured in control and treated (HFD, STZ) mice sciatic nerves by Western blot (**B**), *n* = 4–5. Data are expressed as means ± SEM. ** *p* < 0.01. Representative images of sciatic nerve sections from control and experimentally treated mice, 400× magnification, cross-section stained with 2% osmium tetroxide and 1% toluidine blue (**C**), *n* = 4. Scale bar = 100 μm.

In animals with pharmacologically induced diabetes and HFD-induced prediabetes, the total number of axons in sciatic nerves was markedly reduced (Figure 3A), as compared with control mice (2390.75 ± 90.62 in STZ and 2507.56 ± 110.68 in HFD vs. 3257.44 ± 61.14 in CTRL group, *p* < 0.01). Morphometric data parallel changes observed in the expression levels of β-ACTIN in sciatic nerves: although differences between the means did not reach statistical significance, lower levels of protein expression were detected both in STZ- and HFD-treated mice (Figure 3B; Figure S2 in the supplementary). Moreover, as depicted in Figure 3C, the results of the light photomicrography of sciatic nerves point to an increased irregularity in morphology (e.g., atypical and asymmetrical shape of axonal cell bodies) in most of the nerve fibers comprising the sciatic nerves from both treatment groups (Figure 3C).

### 3.4. STZ/HFD Mice Manifest Impaired Oxidative/Antioxidative Balance in Peripheral Nerves

**Figure 4:**
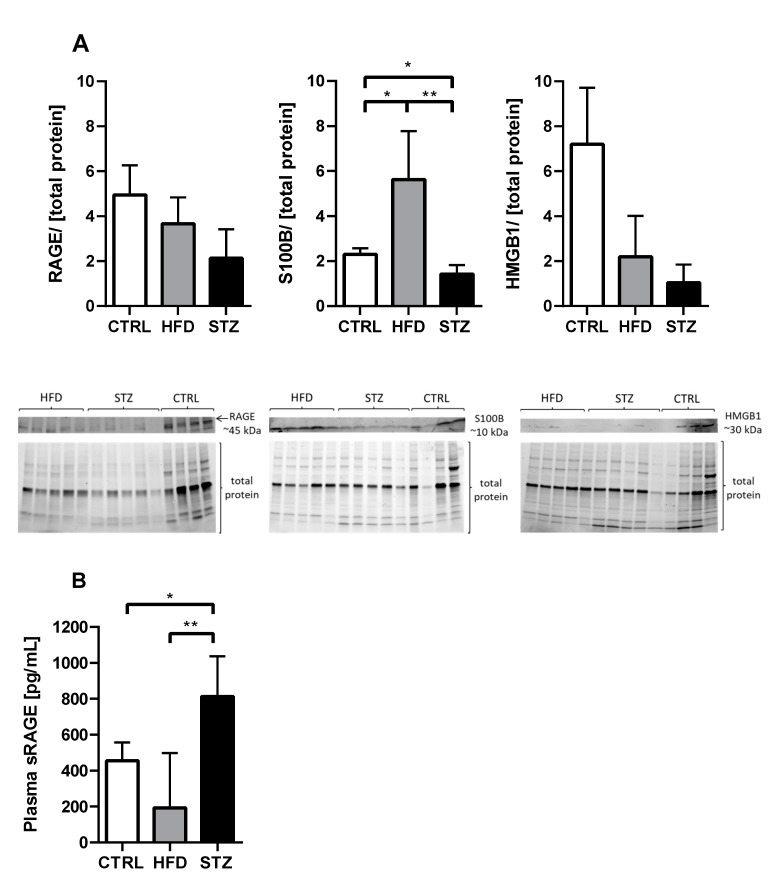
Expression level of proteins involved in neuroinflammatory process is altered in the sciatic nerve and plasma of STZ/HFD mice. Western blot analysis of the proteins involved in neuroinflammation (**A**) measured in control and experimentally treated mice; *n* = 4–5. Circulating level of sRAGE in plasma (**B**); *n* = 6. Data are expressed as means ± SEM. * *p* < 0.05; ** *p* < 0.01.

HFD mice had significantly increased expression levels of S100B protein as compared with control and STZ mice (5.62 ± 0.96 vs. 2.29 ± 0.27, *p* < 0.05 and vs. 1.41 ± 0.18, respectively; *p* < 0.01) (Figure 4A). On the contrary, STZ mice showed a decreased expression of this protein, as compared with CTRL mice (*p* < 0.05). Surprisingly, the levels of expression of RAGE and HMGB1 were decreased in both experimental groups, however, these trends did not reach statistical significance (Figure 4A; Figure S2 in the supplementary). The level of circulating sRAGE in the plasma of STZ-administered mice was significantly higher than in HFD and CTRL mice (812.5 ± 91.56 vs. 192.7 ± 136.6, *p* < 0.01 and 455.0 ± 102.5, *p* < 0.05, respectively) (Figure 4B).

**Figure 5:**
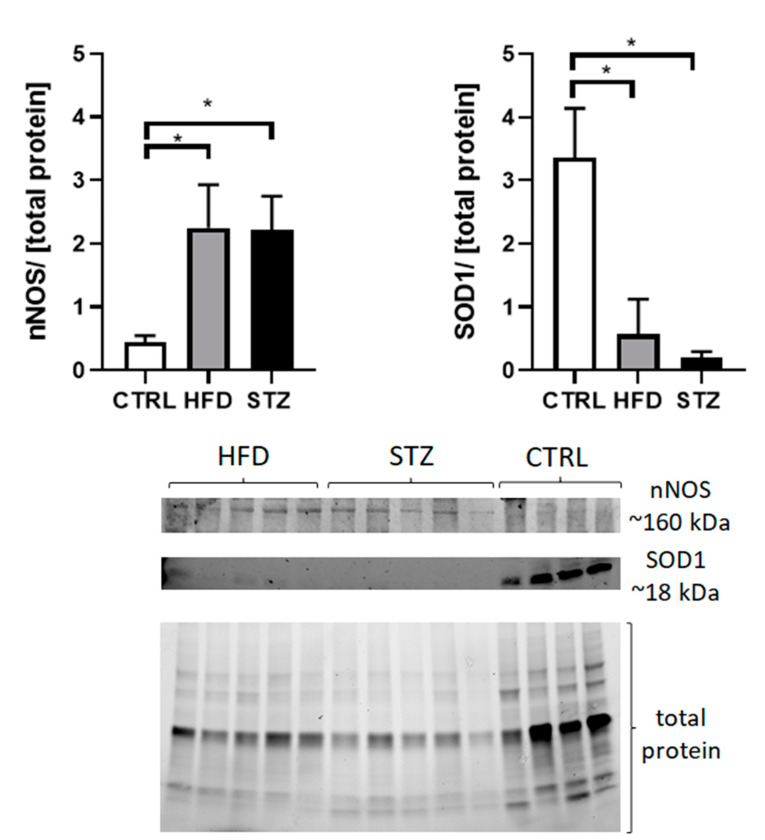
STZ/HFD mice manifest impaired oxidative/antioxidative balance in peripheral nerves. Western blot analysis of proteins involved in oxidative stress/antioxidant defence measured in control and experimentally treated mice. Data are expressed as means ± SEM. * *p* < 0.05; *n* = 4–5.

In comparison with CTRL, both groups of mice receiving experimental treatment had reduced enzymatic antioxidant defence systems, as illustrated by the decreased expression level of superoxide dismutase (SOD1) (3.36 ± 0.77 vs. 0.57 ± 0.24 in HFD-fed and 0.21 ± 0.04 in STZ-treated mice, *p* < 0.05) and increased expression level of neuronal nitric oxide synthase (nNOS) (0.44 ± 0.10 vs. 2.25 ± 0.31 in HFD-fed and 2.22 ± 0.23 in STZ-treated mice, *p* < 0.05) in sciatic nerves (Figure 5; Figure S2 in the supplementary).

The presence and intracellular localization of β-ACTIN, S100B, RAGE, HMGB1, SOD1 and nNOS proteins in axons and Schwann cells of diabetic mouse sciatic nerves was confirmed by IHC staining (Figure S1 in the supplementary). β-ACTIN is visible in both the axons and Schwann cells of the sciatic nerve; S100B, HMGB1 and SOD1 proteins are localized in Schwann cells and RAGE is present along sciatic nerve axons and, most likely, in Schwann cells (the arrows indicate representative staining areas). Finally, nNOS protein is present in a small number of cells in mouse sciatic nerves.

## 4. Discussion

The present study aimed to compare the effects of T1D and T2D on morphological and electrophysiological properties of sciatic nerves in mice. Eight-weeks-old C57BL/6 mice were subjected to either multiple STZ injections (five doses over one week) or long-term HFD-feeding (18 weeks). Unsurprisingly, in the final stage of the experiment, at the age of 26 weeks, STZ-administered mice had fully developed T1D with hyperglycemia, while T2D mice fed high-calorie diet manifested marked obesity and dyslipidemia. The two experimental regimes resulted in a comparable degree of functional and structural peripheral nerve damage, however, we suspect that the course of events preceding the development of the neuropathological changes in the mice was different for each type of treatment.

Clinical and experimental data show that DPN is triggered by multiple interactive pathogenic mechanisms [4,5,6]. Among others, sustained hyperglycemia seems to be a prerequisite for the development of neuropathic conditions. However, previous studies have shown that symptoms of neuropathy in the sciatic nerve were observed even before fully symptomatic diabetes was manifested in obese patients [11,12] and obese animal models [8,13] exhibiting relatively normal blood glucose levels. This notion suggests that the presence of hyperglycemia alone is not a sufficient and indispensable factor in producing the outbreak and progression of nerve damage observed in DPN. Indeed, accumulating evidence points at obesity-driven hyperlipidemia as an important contributor to the development of this condition [7,8,9]. In the current study, HFD-fed mice exhibited blood glucose concentrations within the normal range. Yet, they had elevated blood lipids and displayed neuropathic changes in peripheral nerves corresponding to those observed in the STZ-induced diabetic mouse model, showing substantial hyperglycemia. Sciatic nerves dissected from HFD-fed mice were characterized by a reduced number of axons with changed morphology. Moreover, HFD mice developed noticeable motor and sensory electrophysiological deficits, although differences between the mean values for MNCV and SNCV for healthy controls and obese mice did not attain statistical significance. These data suggest that dyslipidemia and metabolic syndrome in HFD mice may underlie the root cause of neuropathy, independently of glycemic status. 

The study demonstrates that STZ administration and long-term HFD feeding promote oxidative-nitrosative stress, manifested by elevated nNOS and decreased SOD1-proteins expression in peripheral nerves. Systemic and neuronal oxidative-nitrosative stress may be a large component of the pathogenesis of DPN, and points to the contribution of free radicals and oxidants in the development of both nerve-conduction deficits [14,15,16] and morphologic features characteristic of this condition [17]. nNOS is an important producer of nitric oxide (NO), which acts as a neurotransmitter in multiple physiological transduction pathways. However, at high concentrations, NO becomes cytotoxic and, in pathological conditions, nNOS upregulation can yield toxic NO levels [18]. It has been observed that excessive NO production plays an important role in the pathological process of many neurodegenerative disorders, such as ALS, multiple sclerosis, HIV dementia and Alzheimer’s, Parkinson’s and Huntington’s diseases [19]. Moreover, a growing number of studies have revealed that overexpression of nNOS has also been linked with inflammatory and neuropathic pain [20,21,22]. SOD1, on the other hand, is one of the primary intracellular antioxidants, thus, its deficiency diminishes the capacity of a cell to protect against oxidant damage, an event that could trigger apoptosis. SOD1 activity was found to be reduced in peripheral nerves of mice with experimentally induced DPN [16,23]. Our study shows that superoxide excess accompanies detrimental changes in peripheral nerves induced by either an early, pre-diabetic state or prolonged diabetes.

Another substantial mechanism apparently present at the stage of pre-diabetic neuropathy is the stimulation of inflammatory processes in the sciatic nerve, reflected by the elevated expression of the calcium binding protein, S100B in the neural tissue upon HFD feeding to mice. There is evidence that higher concentrations of S100B can cause apoptosis in neurons and promote the secretion of a broad spectrum of pro-inflammatory cytokines by macrophages [24]. S100B is believed to mediate this response through interaction with a cell-bound receptor RAGE. The formation of AGEs as a result of the nonenzymatic glycation and oxidation of proteins, lipids and nucleic acids commonly occurs during prolonged hyperglycemia, but they also tend to accumulate in normoglycemia, in a state of excessive ROS production or inflammation. Heightened RAGE expression has been previously observed in the peripheral nerves of experimentally induced diabetic rodents [25,26,27]. In the present study, surprisingly, although we observed the full repertoire of changes from neuropathy within the peripheral nerves of diabetic mice, we did not discover definite changes in the sciatic nerve protein expression of RAGE or its ligand HMGB1 between experimental treatments. The observed discrepancies in the expression levels of RAGE and its ligands in diabetic mice nerves between our data and most of the published research findings may be influenced by differences in the applied research methodology: we tried to estimate the amount of target proteins by Western blotting, while most scientific works in this field adapt microscopy to visualize and quantify protein expression on immunofluorescent tissue sections of peripheral fibers. In addition, our previous research [26] and current experiences (unpublished), show that different study designs significantly influence data on RAGE protein expression levels, which tends to be more affected when peripheral nerve pathology is a consequence of an acute condition (e.g., nerve injury), rather than long-developing persistent disease. In the mentioned study RAGE and HMGB1 upregulation in wild-type diabetic mice was rather insignificant; only after the crush injury was a marked increase in their expression observed [26]. Noticeably, we detected elevated levels of the secretory isoform of RAGE, sRAGE, in the plasma of STZ-administered mice. Contrary to our observations on RAGE expression in the diabetic peripheral nervous system, several studies show that the blood concentration of sRAGE may reflect tissues’ RAGE expression [28,29]. The exact function of sRAGE is still unknown, however it is presumed to counterbalance the disadvantageous action of the full-length receptor by acting as a decoy and competing with RAGE for ligands [30,31]. Additionally, there is substantial between-study heterogeneity in the serum levels of sRAGE measured in patients with T1D; relative to non-diabetic controls, there are reports of greater [32,33] and also unchanged or even lesser expression [34,35]. Increased sRAGE expression in T1D patients could be a defensive reaction to partly counteract the elevated levels of AGEs. On the other hand, our study detected relatively low levels of circulating sRAGE in the plasma of HFD-fed mice; this was unsurprising, since low levels of this parameter have been previously observed in obese pre-diabetic patients, together with increasing numbers of the components of the metabolic syndrome [36,37]. Accordingly, our findings on the expression changes of RAGE isoforms, both locally in the sciatic nerve and systemically in the blood, do not provide an explanation for the role of RAGE/AGE- mediated processes in the development of symptoms of diabetic neuropathy. Further studies are needed to explore their potential role in the pathomechanism of DPN evolution.

In the present study, STZ mice with long-term experimental diabetes developed changes in the electrophysiology of their sciatic nerves. Pre-diabetic HFD mice also demonstrated evidence of mild electrophysiological deterioration, which is consistent with previous reports [38], yet the NCV values between healthy and obese mice were not statistically different. SNCV and MCNV records provide the most accurate and non-invasive medical procedure measure of subclinical peripheral large, myelinated nerve fiber-impaired functioning. However, NCV is not sensitive enough to capture and assess small sensory and unmyelinated fiber damage. The earliest manifestations of pathological changes in peripheral nerves that are observed in the early pre-diabetic condition mainly occur in small, unmyelinated, or moderately myelinated fibers. It may be the reason why, in our study, NCV deficits in HFD-fed mice were only of limited intensity.

Morphologic and morphometric studies of sciatic nerve semithin cross-sections from STZ and HFD mice revealed axonal loss and additional features pointing to the deterioration of fiber architecture (Figure 3A,C). Neurons isolated from both experimentally treated groups are more often irregular, with examples of axons of asymmetrical structure, demonstrating deviations from a circular shape. Moreover, micrographs show that axons of mice fed an obesogenic diet were more scattered throughout the nerve, with lots of void spaces between the bundles. Of note is an observation that the axonal atrophy seen in diabetic and pre-diabetic mice coincides with a moderate, though statistically insignificant, reduction in the expression and axonal transport of the cytoskeletal protein β-ACTIN in the peripheral nerves of these mice (Figure 3B). Collectively, we suspect that histologic changes found in the peripheral nerves of diabetic and pre-diabetic mice, namely a loss of nerve fibers, considerable fiber shape heterogeneity and the incorporation of numerous non-circular fibers are responsible, at least in part, for the nerve-conduction deficits detected in these mice.

In the present study, STZ-administered diabetic and HFD-fed pre-diabetic mice revealed similar characteristics of peripheral nerve damage, including a number of morphological and electrophysiological pathological changes in the sciatic nerve. Our findings confirm that HFD, prior to the development of fully manifested diabetes, induces neuropathic changes in large nerve fibers of C57BL/6 mice and glycemic status has, in fact, little effect on neuropathy development and progression in T2D. Further studies are needed to investigate the underlying mechanism between HFD and the development of peripheral neuropathy, specifically which abnormalities associated with the metabolic syndrome are the primary drivers of pathological changes in peripheral nerves.

## Data Availability

The data that support the findings of this study are available from the corresponding author upon reasonable request.

## Supplementary Materials

The following are available online at https://www.mdpi.com/article/10.3390/life11111267/s1, Figure S1: The presence and intracellular localization of β-ACTIN, S100B, RAGE, HMGB1, SOD1 and nNOS proteins in a diabetic mouse sciatic nerve. Representative immunohistochemical (IHC) staining of a diabetic mouse sciatic nerve at 400× magnification. Sciatic nerve was stained for β-ACTIN, S100B, RAGE, HMGB1, SOD1 and nNOS. Arrows indicate typical location of a given substance as stained by IHC. NC, negative control. Scale bar = 100 μm; *n* = 4; Figure S2: Western blots and densitometry analysis of β-ACTIN; proteins involved in neuroinflammation (RAGE, HMGB1, S100B) and oxidative stress/antioxidant defence markers (SOD1, nNOS) in the sciatic nerves of control (CTRL) and experimentally treated mice (HFD, STZ). An equal amount of protein (40 µg) was fractionated in a 15-well 4–15% Mini-PROTEAN^®^ TGX™ Precast Protein Gels (Bio-Rad, Hercules, CA, USA) and transferred to nitrocellulose membranes. The bands were visualized with ChemiDoc Imaging Systems (Bio-Rad). Images were quantified densitometrically with ImageJ Software 1.50i (Wayne Rasband, Bethesda, MD, USA) and compared to experimental condition after normalization to the total amount of protein in a sample. The results were expressed in relative folds change.
