# Supplementary material for: Peripheral Neuropathy Presents Similar Symptoms and Pathological Changes in Both High-Fat Diet and Pharmacologically Induced Pre- and Diabetic Mouse Models
Source: Life (Basel). 2021 Nov 19;11(11):1267. doi: 10.3390/life11111267 (PMC8618965; doi:10.3390/life11111267)
Supplement: Supplementary file 1 [file life-11-01267-s001.zip › life-1449007-supplementary.pdf]

life

Supplementary materials

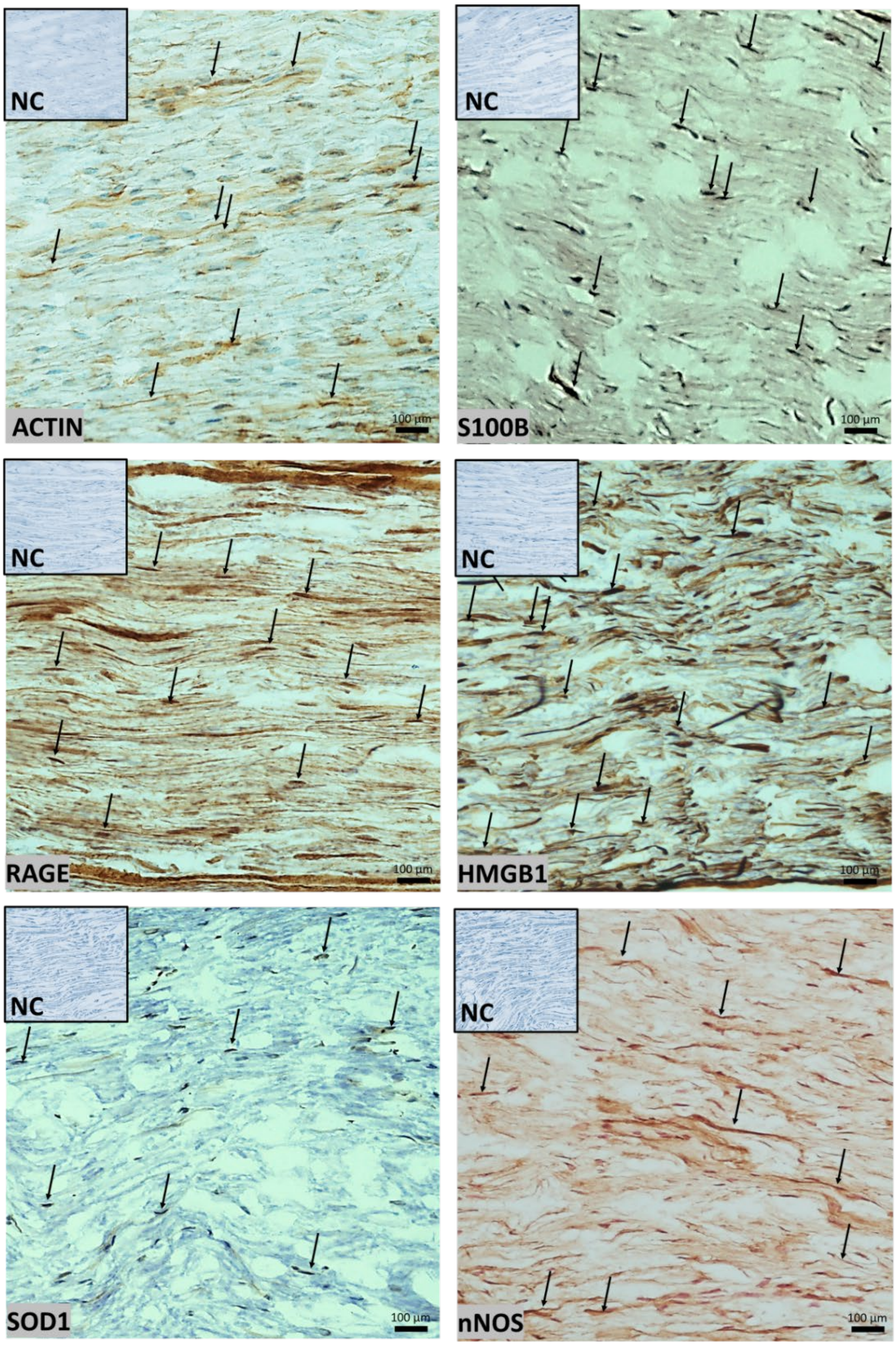

Figure S1. The presence and intracellular localization of β-ACTIN, S100B, RAGE, HMGB1, SOD1 and nNOS proteins in diabetic mouse sciatic nerve. Representative immunohistochemical (IHC) staining of mouse diabetic sciatic nerve with a 400 × magnification. Sciatic nerve was stained for β-ACTIN, S100B, RAGE, HMGB1, SOD1 and nNOS. Arrows indicate typical location of a given substance as stained by IHC. NC, negative control. Scale bar = 100 µm; *n* = 4.

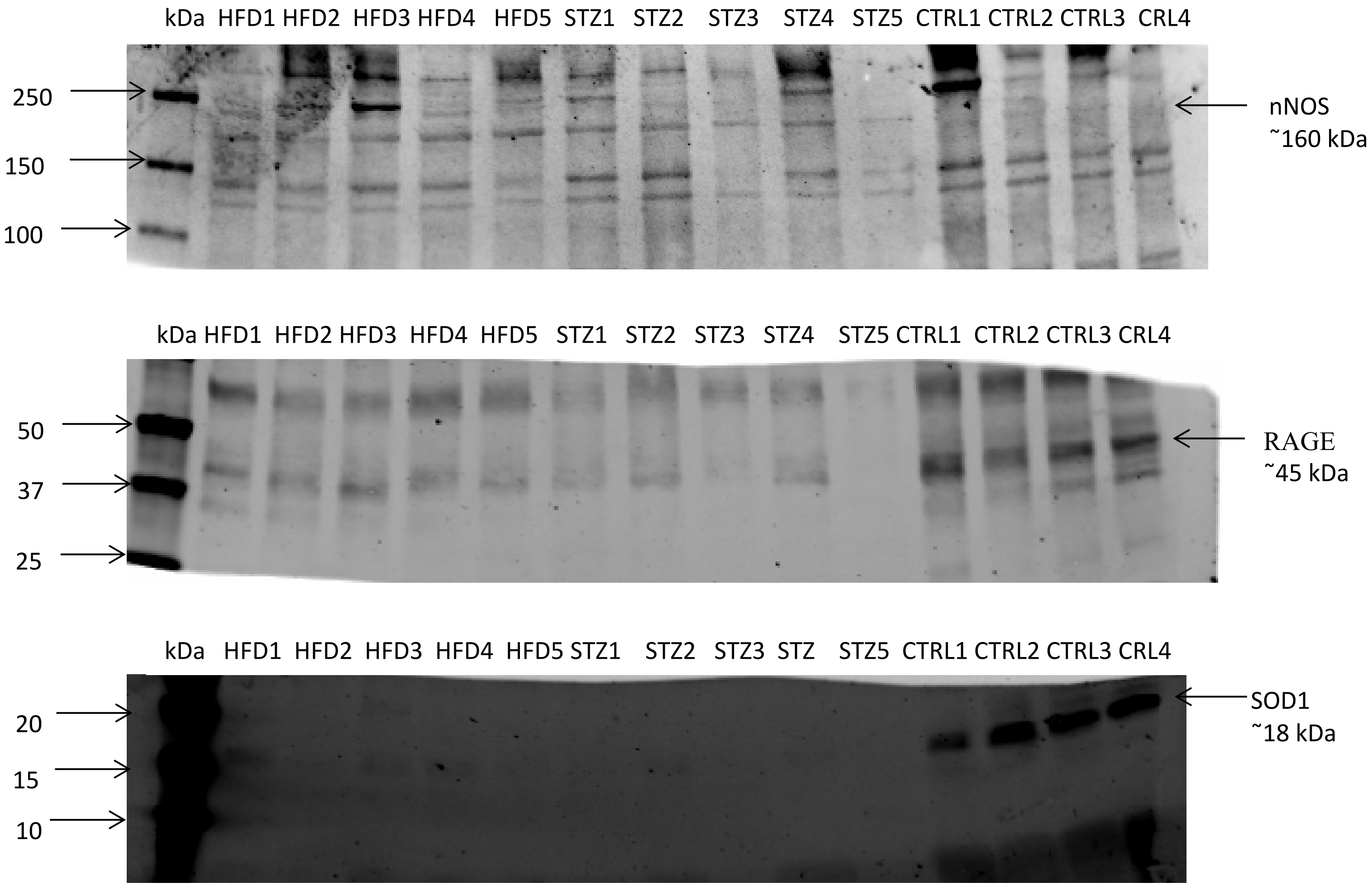

HFD1 HFD2 HFD3 HFD4 HFD5 STZ1 STZ2 STZ3 STZ4 STZ5 CTRL1 CTRL2 CTRL3 CRL4

total
protein

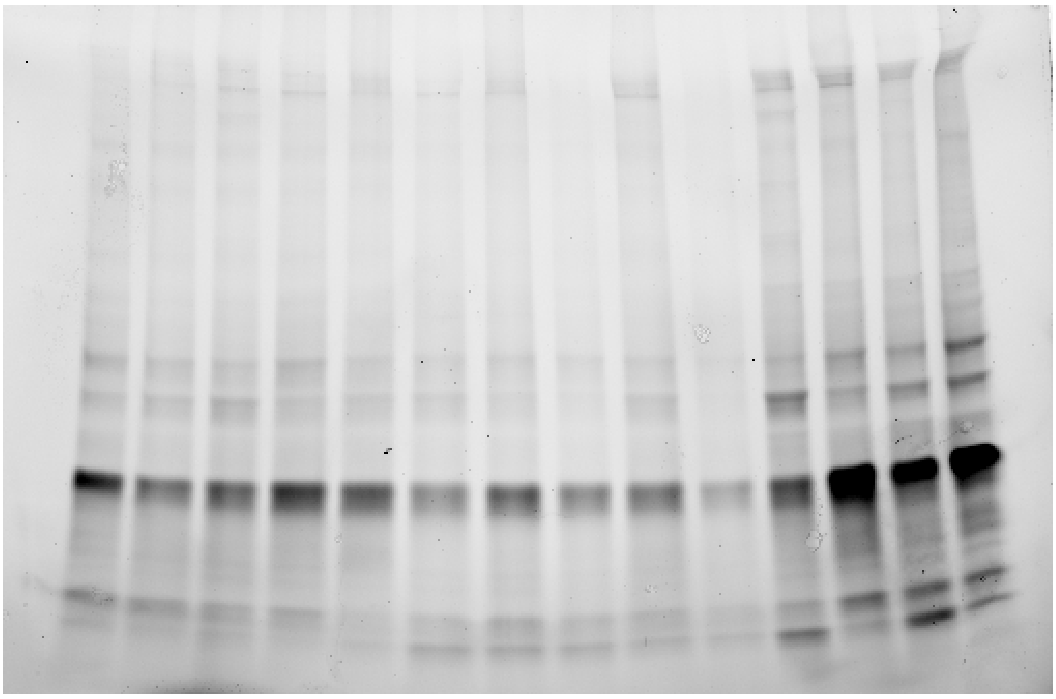

	nNOS/total protein	RAGE/total protein	SOD1/total protein
HFD1	2,3839	3,200309	1,499638
HFD2	1,351527	4,097107	0,642672
HFD3	1,890208	5,502389	0,265383
HFD4	2,404352	2,576262	0,355775
HFD5	3,199574	2,93817	0,103494
STZ1	2,849426	2,887909	0,351274
STZ2	1,530053	2,438737	0,20404
STZ3	1,916563	1,182554	0,182949
STZ4	2,226647	3,657102	0,202894
STZ5	2,589181	0,475946	0,099415
CTRL1	0,168022	8,605212	1,422198
CTRL2	0,457213	2,425196	2,787161
CTRL3	0,511497	4,113867	4,546964
CTRL4	0,646247	4,660315	4,687937

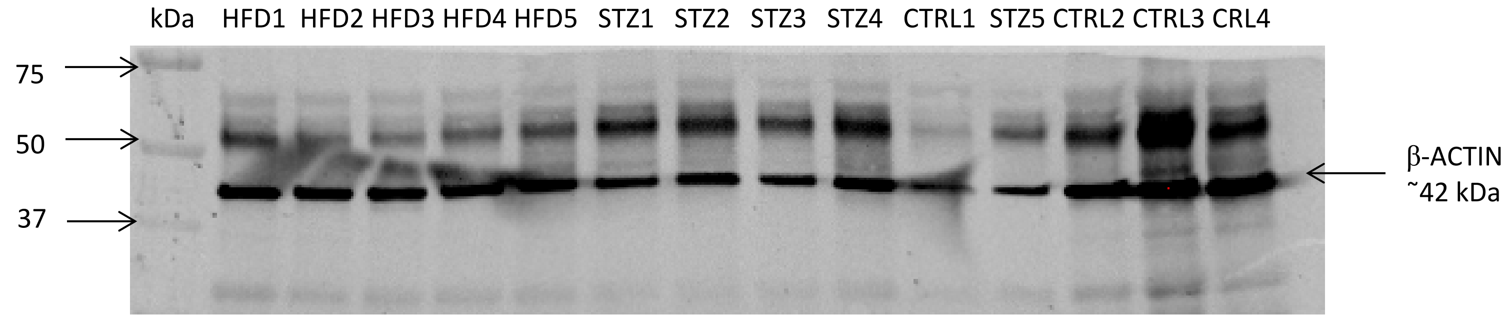

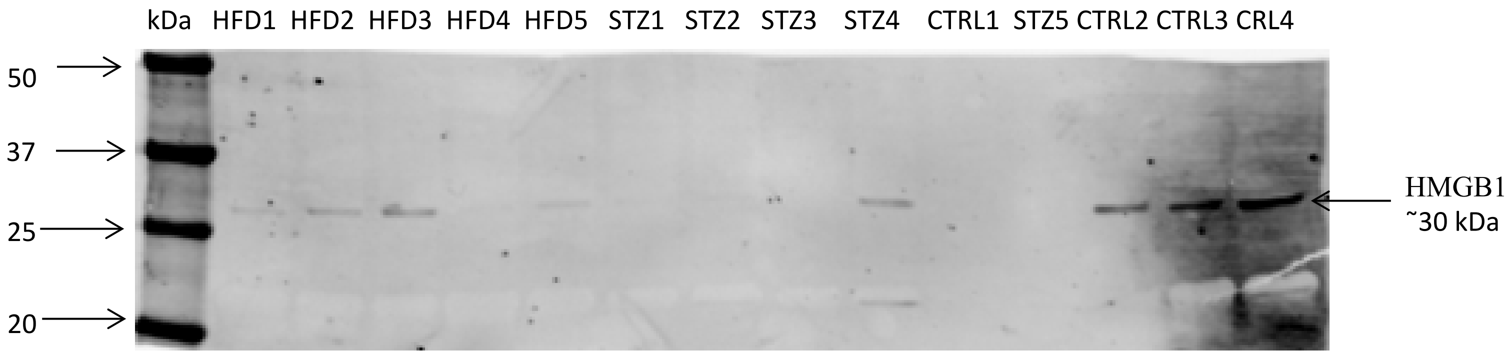

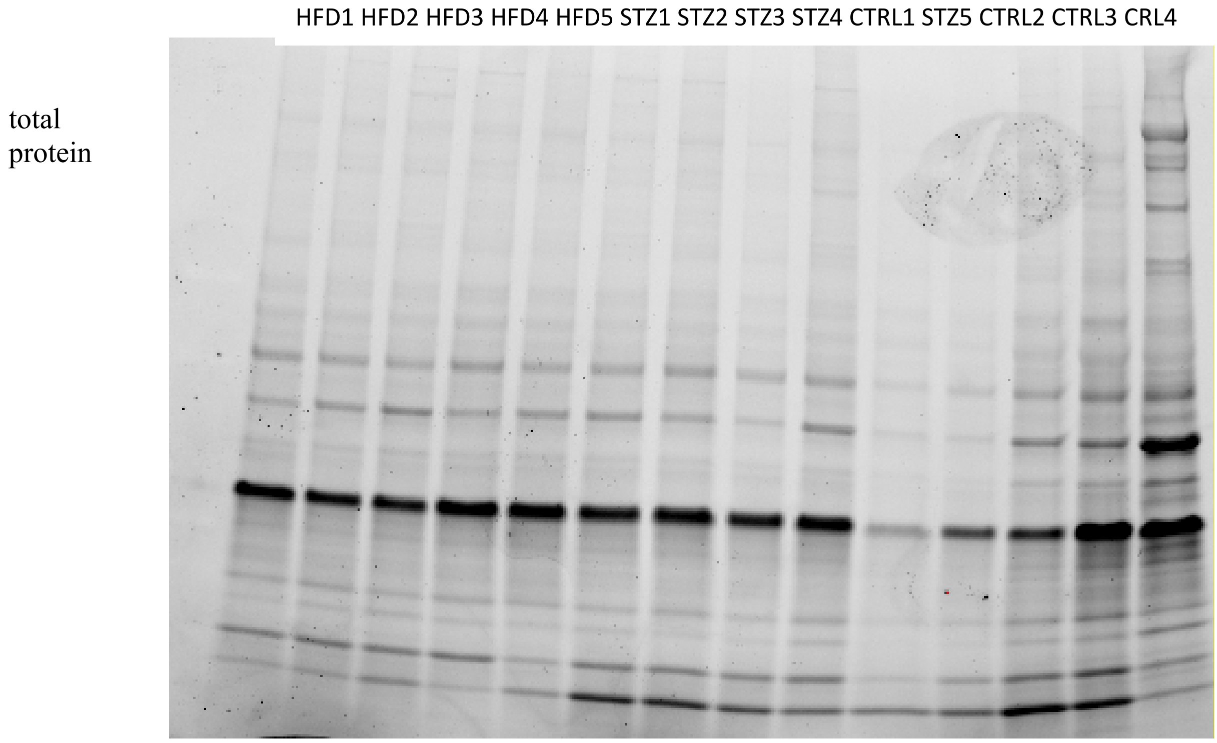

	β-ACTIN/total protein	HMGB1/total protein
HFD1	6,699354	1,39232
HFD2	4,140765	3,72447
HFD3	2,943013	4,539785
HFD4	4,186469	0,533688
HFD5	3,654307	0,793872
STZ1	1,991332	0,304987
STZ2	2,14492	1,239323

STZ3	2,496909	1,09564
STZ4	3,945484	2,263745
CTRL1	3,843156	0,094899
STZ5	3,863384	0,326506
CTRL2	7,302021	10,38958
CTRL3	7,231243	7,191028
CTRL4	2,435065	11,12288

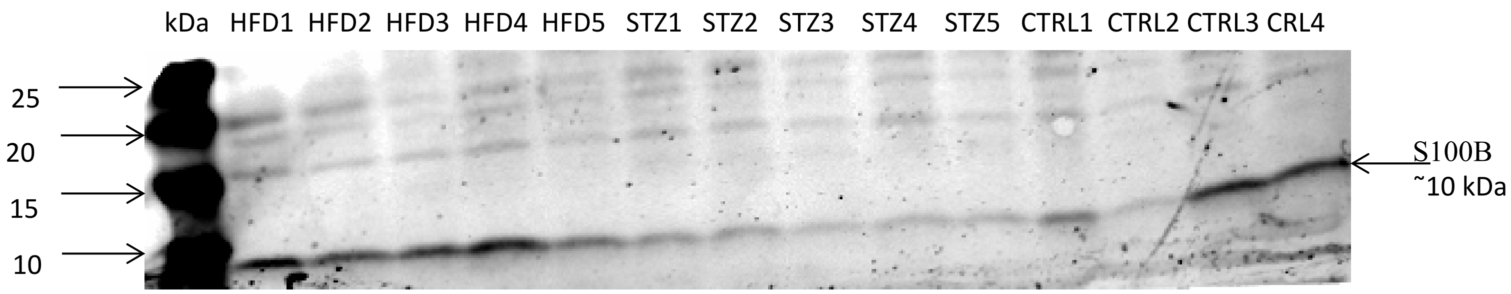

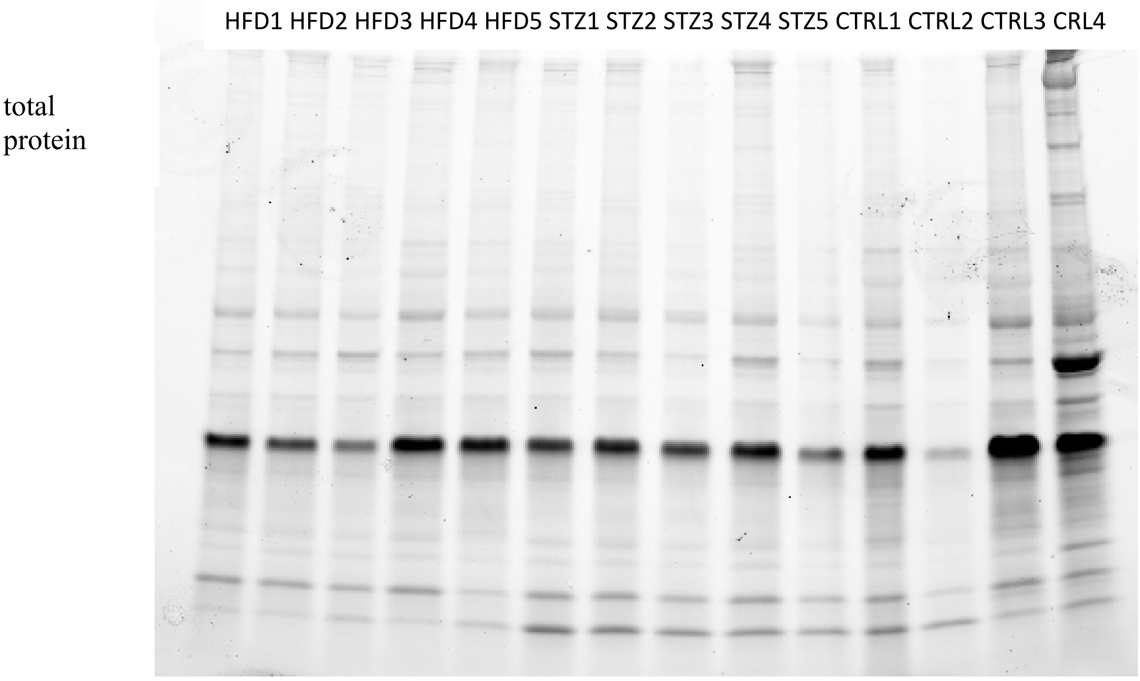

	S100B/total protein
HFD1	8,588954
HFD2	4,375635
HFD3	6,401761
HFD4	5,869355
HFD5	2,878934
STZ1	1,898976

STZ2	1,521093
STZ3	1,039122
STZ4	0,961297
STZ5	1,672953
CTRL1	1,755672
CTRL2	1,918686
CTRL3	2,864447
CTRL4	2,652146

Figure S2. Western blots and densitometry analysis of proteins involved in neuroinflammation (RAGE, HMGB1, S100B), β-ACTIN and oxidative stress/antioxidant defense markers (SOD1, nNOS) in sciatic nerves of control (CTRL) and experimentally treated mice (HFD, STZ). An equal amount of protein (40 μg) was fractionated on 15-well 4–15% Mini-PROTEAN® TGX™ Precast Protein Gels (Bio-Rad, CA, USA) and transferred to nitrocellulose membranes. The bands were visualized with ChemiDoc Imaging Systems (Bio-Rad). Images were quantified densitometrically with ImageJ Software 1.50i (Wayne Rasband, MD, USA) and compared to experimental condition after normalization to the total amount of protein in a sample. The results were expressed in relative folds change.
